# Current intimate partner violence and associated factors among sero-positive women attending Adama town ART Clinics, Central Ethiopia 2019

**DOI:** 10.1186/s12939-022-01647-y

**Published:** 2022-03-31

**Authors:** Girma Garedew Goyomsa, Teklu Arga Albe, Sisay Abebe Debela, Leul Deribe Kitaw

**Affiliations:** 1 College of Health Sciences, Salale University, Fitche, Ethiopia; 2 Department of Population and Family Health, Jimma University, Jimma, Ethiopia; 3 Addis Ababa University, Addis Ababa, Ethiopia

**Keywords:** Sero-positive, Anti-retroviral therapy, Current intimate partner violence

## Abstract

**Background:**

Intimate partner violence against women is a behavior within an intimate relationship that causes physical, sexual, or psychological harm to the victim. It is well recognized as a gross violation of human rights and affects the health of women, families, and the community at large. However, the level to which Human Immuno Deficiency virus sero-positive women are experiencing recent intimate partner violence and its associated factors have not been well investigated as the majority of the study done so far were focused on the study of lifetime violence and violence among women in the general population. The study was conducted to determine the prevalence and factors associated with current intimate partner violence among sero-positive women.

**Methods:**

A facility-based cross-sectional study was conducted from March 2019 to April 2019 among 396 sero-positive women visiting anti-retroviral therapy (ART) units of Adama town public health facilities. A systematic random sampling technique was used to select individual participants. Validated World Health Organization (WHO) tools were used to collect information on the outcomes and key independent variables. The collected data were entered into Epidata version 4.4.6 and analyzed using SPSS version 24. Descriptive statistics were used to compute summary statistics and proportion. Variables at a cut-off value of 0.25 on bivariate analysis and 0.05 during multivariate logistic regression were used to identify factors associated with recent intimate partner violence.

**Result:**

The response rate in this study was 100% since all women approached took part in this study. The prevalence of current intimate partner violence was 32.3% while lifetime intimate partner violence (IPV) was 45.5%. Exposure to coerced first sexual intercourse [AOR = 3.0 (1.73, 5.44)], male multi-partnership [AOR = 2.2 (1.21, 4.06)], believing in the husband's right to sex [AOR = 2.3 (1.29, 4.12)], contraceptive use [AOR = 3.33 (1.67, 6.62)], and having farmer partner [AOR = 3.9 (1.43, 10.79)] were significantly associated with current intimate partner violence.

**Conclusion:**

One-in-three women reported at least 2 or more forms of violence from their intimate partner. Individual-level factors (Exposure to coerced first sexual intercourse, partner’s occupation, contraceptive use, and believing in husband’s right to sex and relationship factor (Male multi-partnership) were significantly associated with recent intimate partner violence. Combined efforts are required to avert intimate partner violence among women on ART while targeting risky sexual behavior practiced among male partner factors significantly associated with violence.

## Background

Intimate partner violence against women (IPVAW) is defined as behavior within an intimate relationship that results in or is likely to result in physical, sexual, or emotional harm to the individual [1]. The United Nations Declaration on the Elimination of Violence against Women (UNDVAW) affirms that violence against women (VAW) constitutes a violation of the rights and fundamental freedoms of women and impairs their enjoyment of those rights and freedoms [2]. IPVAW refers specifically to abuse within an intimate relationship while VAW is broader and encompasses any abuse perpetrated within or outside the family [3]. The occurrences of each form of IPVAW are often characterized by their coexisting nature, i.e., physical IPVAW is often followed by sexual, and it is usually accompanied by psychological violence. In addition, the problem is considered as iceberg phenomenon since it is underestimated and undisclosed especially among sero-positive women [4].

Intimate partner violence against women and human immune deficiency virus/acquired immune deficiency syndrome (HIV/AIDS) is extensively interconnected. Being living with HIV/AIDS increases the risk of violence, and the threat of violence exacerbates the risk of contracting HIV [5, 6]. IPVAW is one of the commonest factors that predisposes women to HIV infection and remains part of their lives after testing positive [7, 8]. Consequently, many women living with HIV regularly face stigma and violence at a greater frequency and severity, making them live poor quality life than HIV negative women [9, 10].

Around 35% of women who experienced violence, 30% of violence is perpetrated by a partner implying that IPVAW is the commonest form of all violence against women [5]. The prevalence of IPVAW among HIV sero-positive women in Africa is among the highest (37%) and its burden is highest in the Democratic Republic of Congo [12]. Although there is no substantial study on partner violence among HIV sero-positive women in Ethiopia, some studies indicate the high burden of reproductive-age women violence. For example, about 20% of reproductive-age women have experienced partner violence, with some studies reporting up to 46% [12, 13].

The effect of IPVAW among sero-positive women is found to be multidimensional, which affects all families by increasing the risk of future ill-health. For instance, fear of new or worsening of existing violence discourages women from disclosing their sero-status to their spouses. This non-disclosure may delay the adoption of safe sex and other preventive practices that could protect the partners and other members of the family, including the unborn baby [14, 15]. The act of IPVAW could affect the mental health of women to make independent decisions about sexual and reproductive health which could protect health and risk of re-infection with a new drug-resistant strain of the virus [16, 17].

Intimate partner violence against women is a barrier to enrollment into the care continuum, i.e., linkage to care, retention in care, adherence to the drug, and viral suppression [18, 19]. Due to the impact of IPVAW, a considerable proportion of HIV sero-positive women failed to initiate treatment and care and are non-adherent to the treatment [20, 21]. For instance, a review and meta-analysis studies suggested that 55% of HIV sero-positive women had lower rates of self-reported treatment adherence [20, 21]. Poor treatment adherence is the most risk of virologic failure (36% decreased odds of viral suppression), low CD4 count, higher incidence of opportunistic infection, high burden of mother to child transmission of HIV (increased infant morbidities), and high mortality rate [19, 22–24].

Due to the complex nature of IPVAW, the United Nations General Assembly (UNGA) recognized IPV as a major obstacle to achieving the 2030 goal of ending the HIV/AIDS epidemic [25–27]. Thus, HIV prevention programs at all levels should focus primarily on halting violence among this population as a strategy to end HIV/AIDS epidemics [25, 28]. Despite the effect of IPVAW on HIV prevention and well-being, there is an evidence gap on the prevalence and forms of violence against women living with HIV as previous studies focus on women in the general population and lifetime violence studies, which could not predict the actual burden of violence after women got infected. Thus, this study was sought to fill evidence gap on the burden and factors that are associated with the current intimate partner violence among HIV sero-positive women in Adama, Ethiopia.

## Method

### Study area and period

**Fig. 1:**
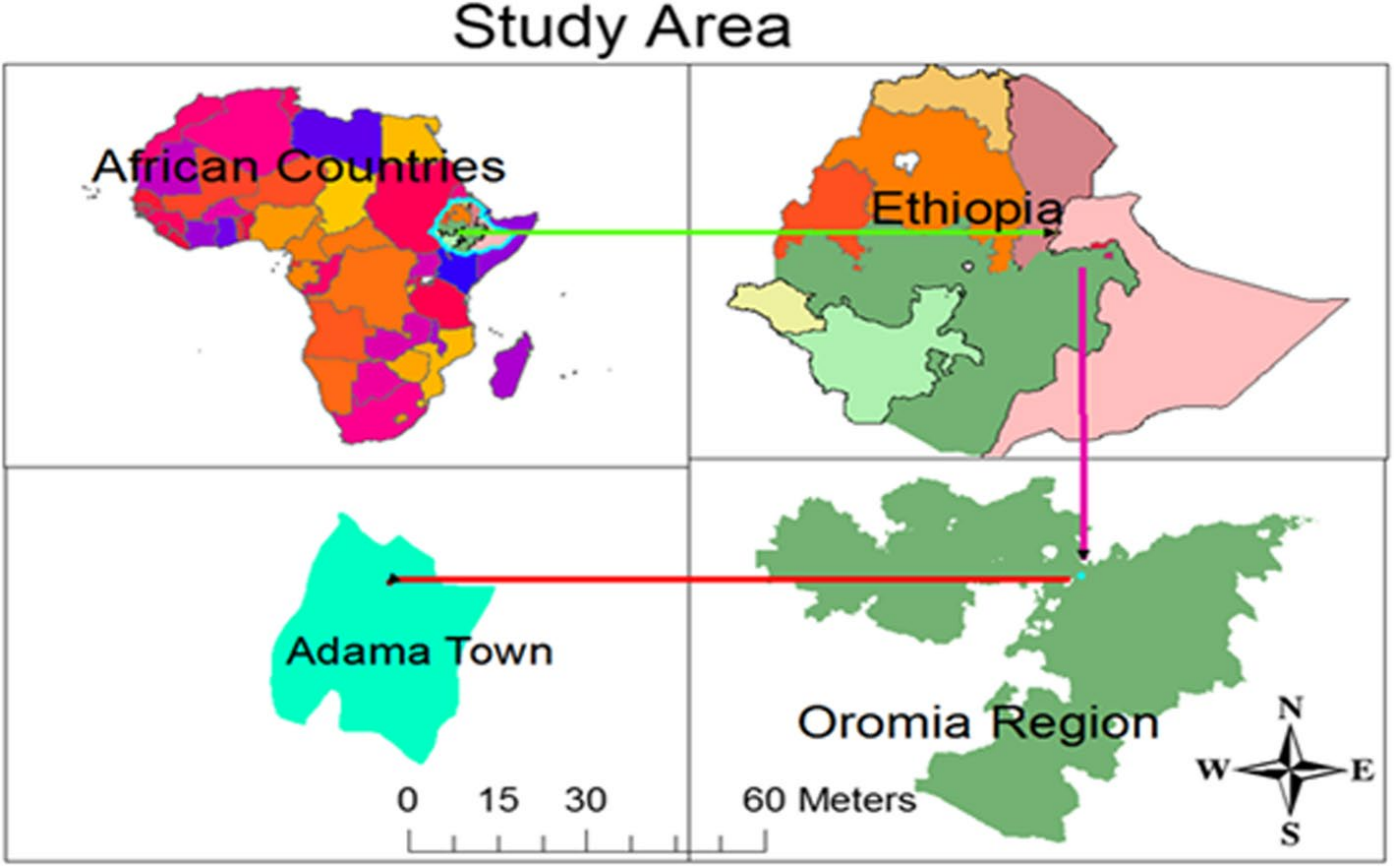
Map of the study area, Adama town, Central Ethiopia, March 2019—April 2019

Adama town (population 220,212) is located in the Oromia regional state of Ethiopia, 99 km southeast of Addis Ababa (Fig. 1). The town has one public hospital and three health centers that provide ART services for 10,944 sero-positive individuals (4517 male and 6427 female) who were older than 15 years. A total of 4072 sero-positive women seek service from the hospital, while 2345 from health centers. The study was conducted at one hospital and three health centers in Adama town from March 2019 to April 2019.

### Study design and procedure

A facility-based cross-sectional study design was employed with a quantitative data collection technique. All sero-positive women older than 15 years and obtaining ART services from public health facilities in Adama town were the source population. HIV sero-positive women older than 15 years with reported history of intimate partner relationships within the past twelve months preceeding the study period were recruited for the interview. The required sample size for the study was determined using a single population proportion formula. Forty-six percent of the true population proportion of violence among sero- positive women (from a previous study [13], 95% confidence level, and 5% estimation precision were considered during the sample size calculation. Accordingly, a total of 382 sample size was determined to be enrolled.

Since the total number of the target population was less than 10,000, the final sample size was corrected for the population and the corrected sample size was 360. The sample size was further inflated by 10% for the non-response rate and the final sample size was 396**.** All public health facilities with ART services (four facilities) (one hospital and three health centers) were included in this study. The total sample size was allocated to the selected health facilities proportionally based on their average number of case flow. Individual participants were approached through calculating sampling interval K [N/n, where N is the total number of women attending ART clinics at the health facilities and n is the final sample size calculated]. Accordingly, the total number of women on ART (*N* = 6427) of whom (*n* = 396) were included in the study yielded a sampling interval of sixteen. The first patient to be interviewed was selected by lottery method among the first sixteen patients. Finally, every sixteen women coming to the ART clinic were included in the study.

### Measures

The outcome variable was a report of current intimate partner violence which was assessed by a cross-culturally validated questionnaire developed by WHO for research on intimate partner violence [29]. A structured questionnaire was initially designed in English and translated into the Amharic language by the translator, and then translated back to English by a third person to check for consistency. The questionnaire had nine sections (socio-demographic, behavioral, general health status, social support, women's attitude toward violence, reproductive history, etc.). Sexual violence was measured using three items (being physically forced to have sexual intercourse against her will from her partner, having sex because she was afraid of what her husband might do, and being forced to do something sexual that she found humiliating or degrading). Six items were used to assess physical violence (slapped or thrown something at her, pushed/shoved her, hit with the fist/thrown something that could hurt, choked/burnt her on purpose, threatened her with/actually used a gun and kicked/beaten up). Four items (insulting, Belittling or humiliating, scaring, and threatening to hurt) were used for emotional violence. Having answered “yes” to at least one item in physical and/or sexual violence within the past twelve months was categorized as women were faced current intimate partner violence.

Perceived social support was measured using a 12-item multidimensional Likert’s scale. The total sum score of the items ranges from 12 to 84 and reliability is between 0.89 and 0.91 by Cronbach’s [30]. Alcohol use for respondents was measured using the alcohol use disorder identification test (AUDIT-C), which comprises three questions, each scored from 0–4, for a total summed score of 0–12. A score of 3 or higher indicates hazardous drinking for women (dichotomized) [31]. Two Bachelor of Science (BSc) nurses who have experience in supervision and four female BSc psychiatry nurses were recruited to collect data from the respondents. The data collection process was taken place in a private room during client exit from the ART unit.

The pre-testing process was conducted on 5% of the total sample size in the adjacent hospital and rigorous training on data collection techniques was given for data collectors to increase data quality. The questionnaire was checked for completeness and consistency by the principal investigator and supervisor.

### Data analysis

After data collection, each questionnaire was checked for completeness and consistency of the information obtained and the data was entered into epidata manager version 4.4.6. Data was exported to SPSS version 24 for cleaning, re-coding, and analysis. Chi-square assumptions were checked before performing bivariate analysis. Multivariate logistic regression at the 95% confidence level was used to identify the predictors. All variables with a *p*-value of ≤ 0.25 were included in the multivariate logistic regression model. A significance level of 0.05 was taken as a cut-off value for all statistical significance tests.

### Ethical consideration

This study was approved by Jimma University, Institute of Health, Faculty of Public Health and medical science ethical review committee. Oral informed consent was obtained from all participants, and all data collection tools were strictly anonymous. The issue of confidentiality throughout the whole process of data collection was discussed and ascertained to the participants.

## Results

### Socio-demographic characteristics

A total of 396 women on ART aged 15 years and older took part in the current study, which gives a 100% response rate. Nearly half (48%) of the respondents were in the age range of 25–34 years with a mean age of 33.4 years ((SD) = 7.1yrs). Oromo were the predominant ethnic group 217 (54.8%) and 226 (57.1%) were Orthodox Christian followers. The mean duration of the relationship with the partners was 10.1 years (SD = 6.5 years) and more than half (57.1%) were in a marital union. Table 1 depicts the socio-demographic of both partners.

**Table 1:** Socio-demographic characteristics of sero-positive women’s attending Adama town ART clinics, Central Ethiopia, 2019 G.C (*n* = 396)

Characteristics	Category	Frequency (*n* = 396)	Percentage (%)
Age	15–24	39	9.9
	25–34	190	48.0
	35–44	136	34.3
	≥ 45	31	7.8
Religion	Orthodox	226	57.1
	Muslim	81	20.4
	Protestant	57	14.4
	Catholic	24	6.1
	Other^a^	8	2.0
Ethnicity	Oromo	217	54.8
	Amhara	104	26.3
	Gurage	40	10.1
	Tigre	32	8.1
	Other^a^	3	0.8
Marital Status	Married	226	57.1
	Cohabiting	85	21.5
	Regular partner & living apart	66	16.7
	Divorced	19	4.8
Respondent Education	No education	181	45.7
	Primary	125	31.6
	Secondary	69	17.4
	Tertiary & above	21	5.3
Respondent occupation	Gov’t employees	56	14.1
	Merchant	99	25.0
	Day laborer	95	24.0
	Farmer	30	7.6
	Housewife	116	29.3
Partner-education	No formal Education	85	21.5
	Primary	131	33.1
	Secondary	111	28.0
	Tertiary & above	69	17.4
Partner occupation	Unemployed	125	31.6
	Farmer	46	11.6
	Merchant	89	22.5
	Gov’t employees	139	34.3
Dowry/ bride price paid	Yes	139	56.7
	No	106	43.3

Other^a^ Wakefata religion, Adare & Hamer ethnicity, Gov’t ^a^ (Government)

### Behavioral characteristics of respondents

The behavioral characteristics of the women and their partners were also assessed. Of all the 396 respondents, 84 (21.2%) reported multi-partnership, and 79 (19.9%) consumed alcohol hazardously. Two hundred three (51.3%) partners of women had a history of multi-partnership relations with another woman and 143 (36.1%) used alcohol daily (Table 2).

**Table 2:** Behavioral characteristic among sero-positive women attending Adama town ART Clinics, Central Ethiopia, 2019 (*n* = 396)

Behavioral characteristics	Category	Frequency (n)	Percentage (%)
Number of partners (current)	Only one	312	78.8
	More than one	84	21.2
Male partner multi-partnership history	Yes	203	51.3
	No	193	48.7
Alcohol consumption	Hazardous	79	19.9
	Non-hazardous	317	80.1
Partner alcohol consumption	Everyday	143	36.1
	1–2 × a week	67	16.9
	1–2 × a month	32	8.1
	Never	154	38.9
Partners involved in fight/riots	Yes	152	38.4
	No	244	61.6
Sero-status disclosure to partner	Yes	183	86.9
	No	28	13.1

Values are presented as frequency and percent

### Reproductive and general health-related characteristics

Of 396 respondents, 126 (31.8%) started sexual intercourse before age 15. The mean age at sexual intercourse initiated was 16.4 years with a standard deviation of (SD = 2.7 years). More than one-third (38.4%) of the women had reported being coerced into the first sexual act. The mean age at which the respondents had known their sero-status was 7.8 years (SD = 3.7yrs). The mean age at which ART initiated was 7.3 years (SD = 3.6 years). About one in four 101 (25.5%) women had changed the anti-retroviral regimen. The major reason for changing regimen was drug side effect 67 (67%) followed by drug resistance 34 (33.7%) (Table 3).

**Table 3:** Reproductive and general health characteristics of sero-positive women attending Adama town ART clinics, Central Ethiopia, 2019 G.C (*n* = 396)

Characteristics	Category	Frequency	Percentage
History of **ever** pregnancy	Yes	319	80.6
	No	77	19.4
Current pregnancy	Yes	34	8.6
	No	362	91.4
Parity	0	45	14.1
	1–4	241	75.3
	≥ 5	34	10.6
Age at sexual debut	< 15	126	31.8
	≥ 15	270	68.2
Condom use (on the recent sexual act)	Yes	183	46.7
	No	211	53.3
History of current use of FP	Yes	199	68.6
	No	91	31.4
Duration since HIV Diagnosis (yrs)	1–2	13	3.3
	2–5	111	28.0
	6–10	122	30.8
	> 10	150	37.9
Aware of partner HIV status	Yes	342	86.4
	No	54	13.6
Sero-status of the partners	Concordant	264	66.7
	Discordant	78	19.3
	Did not know their partner sero status	54	13.6
Viral load	Target detectable	38	9.8
	Target not detectable	358	90.2

-Values are presented as a frequency and percentage

*HIV* Human immunodeficiency virus, *FP* Family planning, *Yrs* Years

### The attitude of the women toward violence

**Fig. 2:**
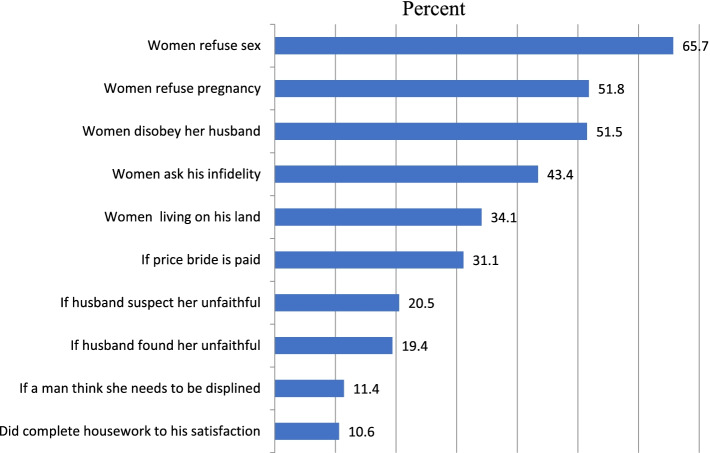
Distribution of justified reason for wife beating among sero-positive women’s attending Adama town ART clinics, Central Ethiopia, 2019

**Fig. 3:**
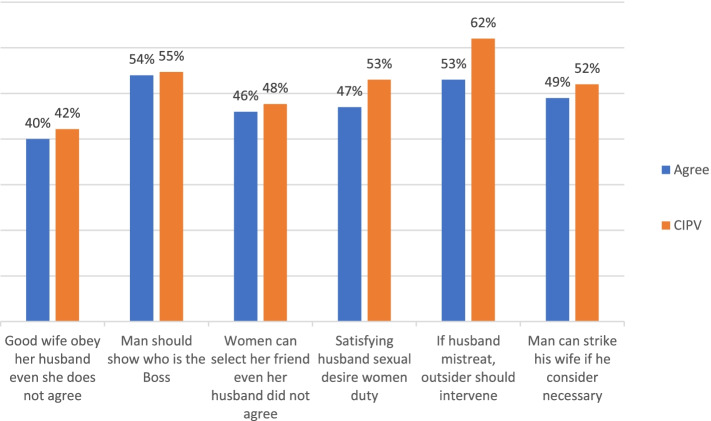
Percentage of women agreed to different gender submissive situation and prevalence of current IPV among sero-positive women’s attending Adama town ART Clinics, Central Ethiopia, 2019

Women`s attitude toward partner violence was also assessed. Accordingly, 80.8% of the women had justified at least one or more reasons that grant a husband the right to violate his partner. The most commonly justified reason was refusing sex (65.7%), refusing pregnancy (51.8%), and disobeying husband (51.5%) (Fig. 2). The distributions of violence in women with a different gender submissive situation were also assessed. Higher violence proportions (62% and 55%) were observed among women who agreed with the statement outsider should intervene if a husband mistreats wife and a man should show who is the boss, respectively (Fig. 3).

### Perceived social support and past history of exposure to non-partner violence

Of the total sample, 185 (46.7%) women had received low social support and only 92(23.2%) received high social support from people surrounding them. Of all the 396 respondents, 160 (40.4%) women reported non-partner physical violence, while 80(20.2%) had reported non-partner sexual violence (Table 4).

**Table 4:** Distribution of perceived social support level and past exposure history to non-partner violence among sero-positive women attending Adama town ART clinics, central Ethiopia 2019, G.C (*n* = 396)

Characteristics	Category	Frequency (n)	Percentage (%)
Perceived social support level of women	Low social support	185	46.7
	Medium social support	119	30.1
	High social support	92	23.2
Exposure to non-partner physical abuse	Yes	160	40.4
	No	236	59.6
Exposure to non partner sexual abuse	Yes	80	20.2
	No	316	79.8
Exposure to family incest (sexual)	Yes	46	11.6
	No	350	88.4

Values are presented as frequency and percent

### Prevalence of intimate partner violence and common types of violence experienced by women in the past year and in their lifetime

The prevalence of current intimate partner violence (physical or sexual/ both) among ever partnered women was 32.3% (95% CI 27.7%, 37.1%), while lifetime violence was 45.5% (95% CI 40.7%, 51%). Current emotional violence was 165 (41.7%), while physical and sexual violence were 107 (27%) and 90 (22.7%), respectively.

Slapping 89 (22.5%) were the commonest reported physical violence which was followed by pushing/pulling hair 64 (16.2%). In most of the respondents, sexual violence which was a result of fear than physical force, and insulting or making her feel bad, 139 (35.1%) were the common emotional acts (Table 5).

**Table 5:** Prevalence rate of different forms and acts of violence among sero-positive women women's attending Adama town ART clinics, central Ethiopia, 2019 G.C

Types of violence	Frequency (*n* = 396)	Percentage (%)
**Physical IPV – at least one act**		
Slapped or threw something at you	89	22.5
Pushed/shoved/pulled her hair	64	16.2
Hitting that could hurt her	46	11.6
Kicked/ beaten her	45	11.4
Choked or burnt you on purpose	10	2.50
Threatened or used a gun/knife	25	6.30
***Total current physical violence***	**107**	**27.0**
**Sexual IPV- at least one act**		
Physically forced you to do sex	55	13.90
Having sex when she didn't want b/c she was afraid of what he may do	73	18.40
Did force you to do something degrading or humiliating	13	3.30
***Total current sexual violence***	**90**	**22.7**
**Emotional IPV –at least one act**		
Insult or made you feel bad about you	139	35.1
Belittled or humiliating you in front of others	67	16.9
Did things to scare or intimidate you purposely	70	17.7
Threatened to hurt you or someone you care	50	12.6
***Total current Emotional violence***	**165**	**41.7**

-Values are presented as frequency and percent (%),—*IPV* intimate partner violence

### Factors associated with intimate partner violence among women on ART

On bivariate logistic regression analysis, thirteen variables were shown an association. Partner’s occupation, respondents & partner alcohol use, male multi-partnership, partner fighting history, contraceptive use, condom use, respondent exposure to physical and sexual non-partner violence after 15 years, and forced first sexual intercourse were found to be associated with the outcome variable. Age at sex initiation, partner substance use, age of the respondent, and believing in the husband's right to sex were significantly associated with the outcome variable.

Multivariate logistic regression analyses revealed that partner occupation [AOR = 3.93 (1.43, 10.79)], male multi-partnership [AOR = 2.21 (1.21, 4.06)], Coerced first sexual intercourse [AOR = 3.06 (1.73, 5.44)], contraceptive use [AOR = 3.33 (1.67, 6.62)], and believe in the husband’s right to sex [AOR = 2.31 (1.29, 4.12)] were significantly associated with intimate partner violence (Table 6).

**Table 6:** Factors associated with current intimate partner violence among sero-positive women’s attending Adama town ART clinics, central Ethiopia, 2019 G.C (*N* = 396)

Variables	Experience of violence			COR (CI 95%)	AOR (95% CI)
	**Yes**		**No**		
Partner occupation					
Unemployed	40 (68.0)		68 (32.0)	1.30 ( 0.76, 2.23)	1.99( 0.97, 4.06)
Farmer	19 (41.3)		27 (58.7)	1.95 ( 0.97, 3.93)^a^	3.93 (1.43, 10.79)^a^
Merchant	33 (37.1)		56 (62.7)	1.63 (0.92, 2.90)	1.81 (0.81, 4.02)
Government employees	36 (26.5)		100 (73.5)	1.00	1.00
Partner engagement in multi-partnership					
Yes	80 (39.4)		123 (60.6)	1.96 ( 1.27, 3.02)^a^	2.21 ( 1.21, 4.06)^a^
No	48 (24.9)		145 (75.1)	1.00	1.00
Contraceptive use					
Yes	66 ( 33.2)		133 (66.8)	2.32 ( 1.25, 4.30)^a^	3.33 (1.67, 6.62)^a^
No	16 ( 17.6)		75 (82.4)	1.00	1.00
Beleiving in a husband’s right to sex					
Agree	69 ( 40.1)		103 (59.9)	1.87 ( 1.22, 2.86)^a^	2.31 (1.29, 4.12)^a^
Disagree	59 (26.3)		165 (73.7)	1.00	1.00
Coerced first sexual intercourse					
Coerced		66 ( 43.4)	86 (56.6)	2.25 ( 1.46, 3.46)^a^	3.0 (1.73, 5.44)^a^
Consented		62 ( 25.4)	182 (74.6)	1.00	1.00
Partner involvement in the fight					
Yes		61(47.7)	91(34.0)	0.56 (0.36, 0.86)	0.55(0.03, 1.02)
No		67(52.3)	177(66.0)	1.00	1.00
Physical violence before age 15					
Yes		58(45.3)	102(38.1)	1.34 (0.88, 2.06)	1.02 (0.53,1.98)
No		70(54.7)	166(61.9)	1.00	1.00
Sexual violence before age 15					
Yes		37(28.9)	43(16.0)	2.1 (1.28, 3.51)	1.88(0.89, 3.95)
No		91(71.1)	225(84.0)	1.0	1.00
Condom use					
Yes	53(41.4)		132(49.3)	0.72 (0.47. 1.11)	0.88(0.48, 1.61)
No	75(58.6)		136(50.7)	1.00	1.00
Age of respondents					
Young	29(22.7)		90(33.6)	0.57 (0.35, 0.94)	0.89(0.46, 1.70)
Older	99(77.3)		178(66.4)	1.00	1.00
Partner drug use history					
Every day	34(26.6)		92(34.3)	0.72 (0.44, 1.19)	0.61(0.30, 1.23)
Once or twice a week	21(16.4)		35(13.1)	1.18 (0.63, 2.18)	1.17(0.46, 2.97)
Once or twice a month	6(4.7)		9(3.4)	1.31 (0.44, 3.84)	0.87 (0.21, 3.67)
Never	67(52.3)		132(49.3)	1.00	1.00
Respondent alcohol intake status					
Hazardous	35 (44.3%)		44 (55.7%)	1.9 (1.15, 3.17)	1.59(0.72, 3.24)
Non-hazardous	93 (29.3%)		224 (70.7%)	1.00	1.00
Partner alcohol intake status					
Never	43 (27.9%)		111 (72.1%)	1.00	1.00
1 to 2 × a week	8 (25%)		24 (75.0%)	1.6 (1.00, 2.72)	1.26(0.63, 2.54)
1 to 2 a month	28 (41.8%)		39 (58.2%)	2 (1.13, 3.86)	0.86(0.32, 2.30)
Daily	49 (34.3)		94 (65.7%)	0.97 (0.40, 2.35)	1.37(0.43, 4.35)

^a^statistically significant at *p* < 0.05 with *cOR* crude odds ratio *aOR* adjusted odds ratio

## Discussion

The present study was aimed to assess the burden of current IPV and factors that predispose to IPV from their current/most recent partner among sero-positive women. The proportion of recent IPV was 32.3% while lifetime IPV was 45.5%. Exposure to coerced first sexual intercourse [AOR = 3.0 (1.73, 5.44)], male multi-partnership [AOR = 2.2 (1.21, 4.06)], believing in the husband's right to sex [AOR = 2.3 (1.29, 4.12)], contraceptive use [AOR = 3.33 (1.67, 6.62)] and having a farmer partner [AOR = 3.9 (1.43, 10.79)] were significantly associated with current intimate partner violence.

IPVAW in the context of HIV is important for both individuals and the wider society; because it contributes significantly to the ongoing HIV transmission. Thus, women who are living with HIV need to be free from any type of violence. However, the current study revealed that the proportion of violence in this population was found to be significant as one in three women reports at least two or more forms of violence from their partner. The proportion of current IPVAW (32.3%) in the present study was comparable with the findings from HIV-positive women reported from Uganda [32] and Nigeria [33]. However, our finding was higher than the finding of a study reported among HIV-positive women in developed countries [34, 35]. This variation could be due to variation in socio-economic status, gender-related health care coverage, and level of law enforcement between our study and the previous studies setup [36]. Moreover, poverty might also predispose women to an increased risk of violence [37, 38]. In addition, low educational attainment could predispose women to an increased chance of violence from their partners. This can be a possible explanation among women in our study where the majority attained lower education and have no job outside the home. Furthermore, the difference between our results and previous study findings might be due to poor commitment to violence reduction intervention at the national and regional levels. For example, universal violence screening service was not started at the national level, and only less effective methods i.e. targeted violence screening service was recently implemented [39–41].

In this study, the prevalence of lifetime intimate partner violence was found to be 45.5%. This figure was in line with the previous findings reported from Ethiopia, which reported it to be 46% [13]. A comparable result of 41% was also reported among pregnant women in the northwestern part of Ethiopia [42]. This indicates that IPV is a common problem for all, irrespective of the women’s health condition. However, our finding was lower than the finding that is reported from HIV-positive women in Kazakhstan (52%) and Togo (63%) [43, 44]. The difference is probably due to the variability in techniques used to measure violence. In the current study, IPVAW was narrowly defined as physical and/or sexual violence by excluding emotional and controlling behaviors. This might be underestimating the proportion of IPVAW compared to previous study findings in which both emotional and control behaviors of violence were included in the definition of IPVAW.

The prevalence of sexual (22%) and emotional violence (41.7%) in the present study were comparable to previous study findings reported among HIV positive women in Ethiopia [13]. Another study conducted among women of the general population in Ethiopia has reported a similar finding on the level of sexual violence (21%) [42]. However, the current finding was higher than the finding of a study reported from HIV-positive women in Uganda in which the proportion of sexual violence was 17.6%, and emotional violence was 17.2% [32]. Another study among HIV positive women from southwest Nigeria indicated lower sexual (2%) and emotional (21%) violence compared to our study finding [45]. The difference between our findings and previous study findings on sexual and emotional violence might be due to variation in the age of women included, where the current study involves women above 15 years, providing good coverage to adequately elicit violence whereas the latter involves only women above eighteen years.

Even though the study was conducted in poor communities which would increase the likelihood of experiencing violence, none of the poverty indicators such as low education and income were significantly associated with IPVAW in the present study. The finding is inconsistent with the findings from a similar study reports from low industrialized, less educated, and poor communities in which low educational status and low economic status were significantly associated with IPVAW in Nigeria [32], Togo [42], and Uganda [31]. This inconsistency might be due to differences in the method used to measure poverty indicators.

The present study suggested that women-related factors such as forced first sexual intercourse were significantly associated with violence. This finding is in agreement with the findings of the study reported from Togo [43]. This could be explained by exposure to first forced sexual intercourse leads to familiarity with sexual violence that women consider normal. These conditions could increase the risk of women’s exposure to violence in their later life and lead to a vicious cycle of violence. Moreover, women living with men who use coercive sexual tactics are more likely to engage in sexual and physical violation [46, 47]. This may indicate the need to foster women's empowerment and social awareness of negative attitudes that reinforces violence against women.

A woman who believes in her husband’s right to sex was more likely to experience violence from her partner. This finding was similar to the study findings from Togo [43] and Ethiopia [13] in which believing in husband's right to sex among women can introduce or escalate the level of violence. This indirectly implies the agreement of women with violent supportive ideas and practices that reinforce their subordination. This makes the women accept violence as a normal condition and leads them to experience violence throughout their lifetime. On the other hand, women with such behaviors are more likely to adapt to live with abusive partners and could be more likely to be exposed to violence than those who did not justify as they may terminate the relationship [48, 49]. This suggests challenging the prevailing gender stereotype in the community is important in reversing the problem.

Contraceptive use was found to increase the chance of violation by three times from their partner. This finding was similar to the findings of the studies reported from HIV-positive women in Kenya [50] and Zambia [51] in which women who tried to negotiate contraceptive use are at increased risk of violence. This could arise from the disagreement between partners on fertility desire where male partners desire having more children [52–54]. Further, low awareness related to contraceptive use among male partners could lead to a violation. This is an indicator for strong attention to couple-oriented reproductive health counseling services at the health facility. Even though contraceptive use was a predictor of IPVAW among sero-positive women, previous literature among women of the general population has not found it to be a predictor of IPVAW [55–57]. This indicates further strong research is required to improve the understanding of dynamics in relationships of IPVAW among HIV positive women and contraceptive use.

Reported multi-partner relationships were significantly associated with a higher proportion of IPVAW. This result was similar to the previous findings from Africa [43, 44, 58–63]. This may be explained by the fact that certain communities acknowledge men having multiple female partners confessed that being questioned about their fidelity could trigger violent acts out of jealousy against female partners [43, 64]. Norms in many underdeveloped countries expect masculine men to be in control of women, and this control can take the form of sexual multi-partnership and violent acts. On the other hand, the prevailing idea of femininity may prevent women from refusing these sociocultural patterns, and on the contrary, seems to promote the acceptance of this behavior, which increases woman's chance of exposure to violence [65, 66]. Effort should target toward tackling of masculinity and feminity constructs in the community.

Husband occupation status was significantly associated with violence in the present study. A woman whose partner is a farmer is more likely to experience violence than the woman who has a government employee husband. This finding was consistent with the finding of a study reported from Nigeria [45]. This might be due to the differences in the level of education attained and level of access to information on gender equality as most commonly, farmers attain lower education and low access rate of information related to the violation which could increase the risk of involvement in abusive behaviors.

### Limitations

The findings of the present study would be interpreted within the context of the following limitations: The assessment of IPVAW was based on a past year time frame which might result in underreporting of violence. Moreover, the experience of IPVAW was based on self-report information which could introduce information bias. However, to minimize information bias, women were given sufficient time to recall their experience and provided multi-option responses to assist in recalling. In addition, some variables such as age at sexual initiation, number of partners, and question used to elicit sexual violence from a respondent were sensitive in their nature and might result in under-reporting of violence. Indeed, to reduce the problem, standardized world health organization questionnaires on IPVAW study and well-trained female data collectors were used to increase information disclosure.

## Conclusions

Intimate partner violence among women on ART was significantly high and public health problems. Individual-level factors, such as justifying violence as a normal, exposure to forced first sexual intercourse, and partner occupation were significantly determined violence. Relationship factor (male multi-partnership) was also significantly associated with violence. Even though, this variable had also shown an association among women in the general population, contraceptive use appears as a unique predictor of IPVAW among HIV positive women. Thus, combined efforts are required to avert IPV among women on ART while targeting risky sexual behavior practiced among partners and other significantly associated factors. In addition, further analysis using qualitative and longitudinal study is required to clearly understand the relationship between violence and contraceptive use among sero-positive women.

## Data Availability

The data set analyzed during the current study will be available from the corresponding author on reasonable request.

## Abbreviations

USAID: United States Agency for International Development
UNICEF: United Nations Children’s Fund
MOE: Ministry of Education
WHO: World Health Organization
IPVAW: Intimate partner violence against women
SPSS: Statistical Package of Social Science
CI: Confidence interval
COR: Crude odds ratio
AOR: Adjusted odds ratio
AAU: Addis Ababa University
IPV: Intimate partner violence
VAW: Violence against women
